# Developments in Nanostructured MoS_2_-Decorated Reduced Graphene Oxide Composite Aerogel as an Electrocatalyst for the Hydrogen Evolution Reaction

**DOI:** 10.3390/gels10090558

**Published:** 2024-08-28

**Authors:** Sadhasivam Thangarasu, Mrunal Bhosale, Gowthami Palanisamy, Tae Hwan Oh

**Affiliations:** School of Chemical Engineering, Yeungnam University, Gyeongsan 38541, Republic of Korea; mrunal.snst.1@gmail.com (M.B.); gowthu.bionat@gmail.com (G.P.)

**Keywords:** hydrogen energy, hydrogen evolution reaction, oxygen evolution reaction, water electrolysis, electrocatalyst, aerogel, hydrogel, MoS_2_, carbonaceous structure, porous structure

## Abstract

Developing lightweight, highly active surfaces with a high level of performance and great stability is crucial for ensuring the dependability of energy harvesting and conversion devices. Aerogel-based electrocatalysts are an efficient option for electrocatalytic hydrogen production because of their numerous benefits, such as their compatibility with interface engineering and their porous architecture. Herein, we report on the facile synthesis of a nanorod-like molybdenum sulfide–reduced graphene oxide (M-rG) aerogel as an electrocatalyst for the hydrogen evolution reaction (HER). The 3D architecture of the network-like structure of the M-rG hybrid aerogel was created via the hydrothermal technique, using a saturated NaCl solution-assisted process, where the MoS_2_ was homogeneously incorporated within the interconnected rGO aerogel. The optimized M-rG-300 aerogel electrocatalyst had a significantly decreased overpotential of 112 mV at 10 mA/cm^2^ for the HER in alkaline conditions. The M-rG-300 also showed a higher level of reliability. The remarkable efficiency of the HER involving the M-rG-300 is principally attributed to the excellent connectivity between the rGO and MoS_2_ in the aerogel structure. The efficient interconnection influenced the achievement of a larger electrochemically active surface area, increased electrical conductivity, and the exposure of more active sites for the HER. Furthermore, the creation of a synergistic effect in the M-rG-300 aerogel is the most probable mechanism to boost the electrocatalytic activity.

## 1. Introduction

In recent years, the energy sector has garnered a significant amount of interest among the different research and development fields due to the depletion of fossil fuels and the environmental problems that are associated with fossil fuels [1,2,3]. Moreover, the continuous increase in the world population is the primary factor driving the need for energy [4,5,6]. Therefore, energy utilization has significantly increased in comparison to previous levels, with fossil fuels being the primary source of energy [5,7]. The usage of fossil fuels releases greenhouse gasses, which have a substantial impact on both the environment and human health [8,9]. In order to address and find solutions to the problems that are associated with fossil fuels, researchers worldwide are inventing various types of energy conversion and storage systems [10,11,12,13,14,15]. For instance, hydrogen energy has garnered a significant amount of interest due to its high energy density (~142 MJ Kg^−1^), as well as the ability to create such energy in an environmentally friendly manner [16,17,18]. The production of hydrogen may be accomplished via a variety of methods [19,20]. Among the several methods of producing hydrogen, the use of water electrolysis for electrocatalytic hydrogen production (2H_2_O → 2H_2_ + O_2_) has garnered much attention due to its environmentally friendly nature, since it produces hydrogen without any carbon emissions (net-zero carbon emissions) [21,22]. According to the electrochemical method, the electrolysis of water is primarily dependent on the activity of the electrocatalyst material. The electrocatalyst material is responsible for effectively dissociating the water molecules into hydrogen and oxygen gasses through the hydrogen evolution reaction (HER) and the oxygen evolution reaction (OER), respectively. Platinum (Pt) is the optimal option for electrode material in the context of the HER, due to its exceptional capacity to facilitate hydrogen evolution and its minimal onset overpotential [16,23,24]. On the other hand, the most significant downsides of Pt-based electrocatalyst materials are their high cost and rare availability [25,26]. Consequently, researchers across the globe have concentrated on creating various types of economically efficient and readily available electrocatalyst materials [27].

Electrocatalyst materials must exhibit both an efficient HER activity and an excellent stability. Therefore, significant attention has been directed at using transition metal-based oxides, carbides, nitrides, sulfides, and phosphides as electrocatalysts to achieve exceptional performance in terms of the HER [28]. The transition metal dichalcogenides (TMDs) category of electrocatalysts has garnered significant interest due to its exceptional activity in this context, its capacity to be adjusted depending on the structural features, and its chemical stability [29,30,31]. Molybdenum disulfide (MoS_2_) has proven its versatility as a valuable material in many applications due to its effective non-toxicity, mechanical strength, physical attributes, and chemical qualities [32,33,34,35]. Micro and nanostructured MoS_2_ have been widely recognized as excellent electrocatalyst materials for the electrocatalytic HER process because of their positive attributes, including their cost-effectiveness, stability, and the presence of abundant active sites. In particular, they exhibit a Pt-like electrocatalyst activity [33]. However, the electrocatalytic activity of bulk MoS_2_ and MoS_2_ alone is not substantial in most cases because of the catalytically inert basal plane [33,36]. MoS_2_ showed an HER overpotential of 205.5 mV [37] and 350 mV [38], at a 10 mA cm^−2^ current density in 0.5 M H_2_SO_4_. In alkaline conditions (1 M KOH), MoS_2_ exhibited an HER overpotential of 234 mV [39] and 239 mV [40] at a current density of 10 mA cm^−2^. Nevertheless, the performance of the MoS_2_ electrocatalyst was significantly enhanced after its modification and the creation of a composite with other materials. The advancements in MoS_2_ research have mostly concentrated on modifying its electrical and structural properties. These modifications include altering the edges, introducing vacancies, doping, inducing phase transitions, its combination with other materials, and alloying [34].

The incorporation of MoS_2_ as a composite electrocatalyst in conjunction with various carbon architectures resulted in a significant improvement in their HER performances [41,42,43]. The composite of reduced graphene oxide (rGO) and MoS_2_ exhibited notable electrochemical activity due to the synergistic effect, whereby the catalytic activity of MoS_2_ was enhanced by the efficient electrical conductivity of rGO [44,45]. Furthermore, oxygen-containing functional groups in the GO facilitate the effective dispersion of nanomaterials, such as MoS_2_. In order to further enhance the performance of typical composite electrocatalyst materials, it is necessary to overcome the inherent difficulties, such as aggregation, lower specific surface area, higher density, non-adjustable porosity, and inferior flexibility. In the recent period, rGO-based aerogels have gained considerable attention in various kinds of energy conversion and storage systems, including water-spitting applications [46,47]. The three-dimensional (3D) porous structure of interconnected network-like rGO aerogels can provide excellent benefits, specifically a high specific surface area and a light density [48]. Hence, the aerogels can significantly impact the HER performances by enhancing electrical conductivity, increasing the abundance of active sites, and facilitating diffusion paths. The 2D layered structure, large surface area, and presence of oxygen containing functional groups in GO can effectively interact with different elements and compounds, which helps form the hybrid rGO-based aerogel formation. A few investigations have been published on MoS_2_-containing rGO hybrid aerogels. In addition to edge modification, vacancy, doping, phase transition, combination, and alloying, the physical characteristics and electrocatalytic performance of MoS_2_ may be adjusted by altering its dimensionalities and crystallinity. From this perspective, we created a nanorod-like structure of MoS_2_ combined with GO to form a MoS_2_-rGO hybrid aerogel. The hydrothermal approach was employed to create a porous 3D MoS_2_-rGO hybrid aerogel using a saturated NaCl solution. Optimal aerogel formation was achieved by comparing two distinct concentrations of rGO and MoS_2_. The MoS_2_ was homogeneously interconnected and incorporated within the network-like structure of the rGO aerogel. The sophisticated structural and microstructural analyses confirm the homogeneity and successful formation of the aerogel. Compared to M-rG-200, M-rG-300 demonstrated an exceptional ability to create aerogels. The M-rG-300 aerogel had an exceptional formation and displayed efficient HER characteristics. Specifically, the M-rG-300 aerogel exhibited a lower overpotential of 112 mV at a current density of 10 mA/cm^2^. The outstanding performance of the HER in M-rG-300 is primarily achieved through the exceptional interconnection between rGO and MoS_2_ in the aerogel. This interconnection influences the increase in specific surface area, electrochemical active surface area, electrical conductivity, and the exposure of higher active sites. In addition, the M-rG-300 aerogel electrocatalyst demonstrated comparable stability.

## 2. Results and Discussion

**Figure 1:**
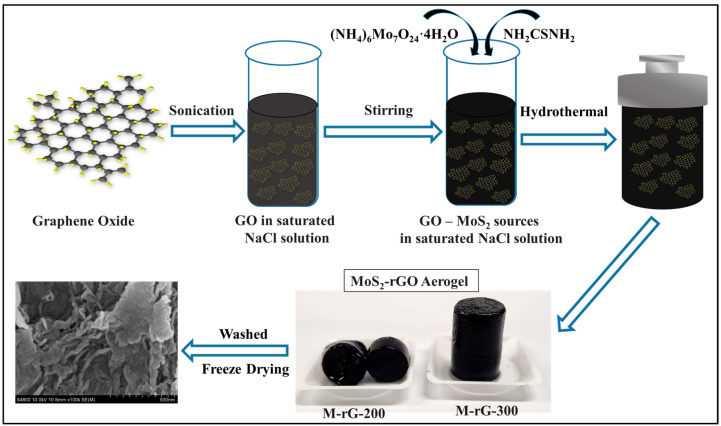
Schematic representation of MoS_2_-rGO aerogel preparation steps.

The synthesis of the aerogel was accomplished by a process consisting of the following two stages: (i) the preparation of graphene oxide (GO) from graphite and (ii) the formation of the MoS_2_-rGO hybrid aerogel (M-rG aerogel). Initially, the graphite acquired in bulk form was subjected to ball milling to decrease its size and number of layers. To effectively complete the development of GO, an improved GO synthesis process was followed [49]. It was decided to utilize a saturated solution of NaCl to develop the nanorod-like structure of MoS_2_ [50] in the rGO aerogel. The overall synthesis process of the M-rG aerogel is schematically explained step-by-step in Figure 1. In order to devolve the M-rG aerogel, a probe sonication approach was used to disperse an expected quantity of GO (200 or 300 mg) in a solution of saturated NaCl. The aerogels, M-rG-200 and M-rG-300, are designated based on the amount of GO in the reaction. The aerogel was produced using a hydrothermal method after the sources used for producing the MoS_2_ were incorporated into the GO solution. To complete the hydrothermal process, the reaction was kept at 80 °C for 30 min at first and then at 180 °C for 24 h. Following the completion of the hydrothermal reaction, aerogels M-rG-200 and M-rG-300 were obtained. It was noticed that the M-rG-200 had been broken into pieces, indicating that it was not produced successfully. On the other hand, a well-structured aerogel with a three-dimensional configuration was successfully formed using M-rG-300. The synthesized aerogel was meticulously subjected to diH_2_O for solvent exchange. Finally, the aerogel in its prepared state was freeze-dried and used for further investigations.

**Figure 2:**
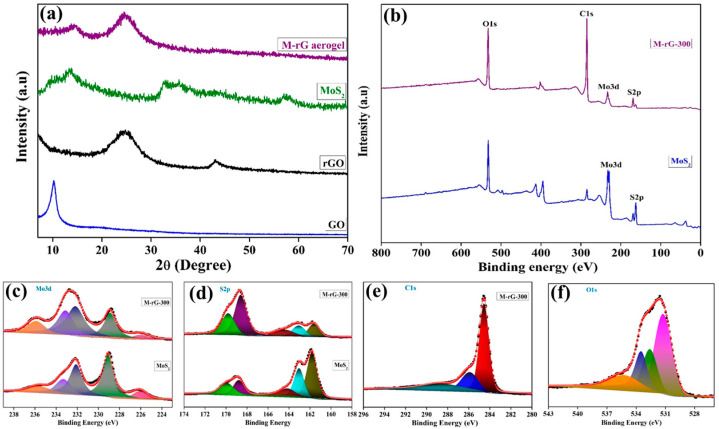
(**a**) XRD spectra of as-prepared GO, rGO, and M-rG-300. (**b**) XPS survey spectra of MoS_2_ and M-rG-300. Deconvoluted XPS spectra: (**c**) Mo 3d, (**d**) S 2p, (**e**) C 1s, and (**f**) O 1s.

The crystalline structure of the as-prepared materials and aerogel was analyzed using X-ray diffraction (XRD). The XRD analyses of GO, rGO, and the M-rG-300 aerogel are shown in Figure 2a. The GO sample exhibited a prominent intensity peak (2θ=) at 10.21°, which corresponds to the lattice plane (001) [51,52]. The XRD data demonstrate the effective preparation of GO by the efficient oxidation of graphite via an improved synthesis method. In the instance of rGO, it has been observed that a peak shift takes place in the GO after the hydrothermal reaction. The largest intensity peaks detected for the rGO were (2θ=) 24.8°, which correspond to the lattice plan of (002) [51,52]. During the hydrothermal process, the evolution of rGO is a sequential process that involves the reduction of oxygen-containing functional groups in GO. For MoS_2_, the diffraction peaks observed at 13.82°, 33.12°, 40.4°, and 57.8° correspond to the (002), (100), (103), and (110) plans, respectively. Moreover, the additional peaks positioned at nearly 9.26° confirm the increase in interlayer spacing for nanorod-like MoS_2_, and the peak at 18.46° reveals the emergence of a second-order diffraction peak [50]. In the case of the M-rG-300 aerogel, the peaks associated with rGO and MoS_2_ were seen to coexist in the XRD spectrum. The XRD results indicate that the MoS_2_ integrated into the rGO and dispersed evenly throughout the aerogel. X-ray photoelectron spectroscopy (XPS) analysis was carried out to further determine the chemical states and elemental compositions in the M-rG-300 aerogel for further identification. Figure 2b displays the entire survey spectra of the XPS recorded of the M-rG-300 aerogel. The XPS analysis indicated that the M-rG-300 aerogel was composed of carbon, oxygen, molybdenum, and sulfur elements. The XPS Mo 3d spectra (Figure 2c) in the M-rG-300 aerogel exhibited two separate peaks at 232.78 eV and 228.78 eV, which corresponded to Mo 3d_3/2_ and Mo 3d_5/2_, respectively [53,54,55]. The deconvoluted spectra reveal distinct peaks with binding energies of 228.88 eV, 232.12 eV, and 233.15 eV, which are indicative of the presence of Mo^4+^ and provide evidence for the existence of mixed 1T/2H MoS_2_ [54]. Moreover, the additional peak on the Mo 3d spectrum is at 226.08 eV, corresponding to S2s. The S 2p spectra (Figure 2d) of the M-rG-300 aerogel show peaks at binding energies of 161.68 eV, 163.08 eV, and 164.38 eV, which correspond to the S^2−^ states of 2p_1/2_, 2p_3/2_, and 2p_1/2_, respectively [56]. The additional peaks at binding energies of 168.58 eV and 169.78 eV correspond to the S-O band [56,57]. The XPS spectra of C1s that have been deconvoluted are shown in Figure 2e. The deconvoluted peaks appeared at 284.58 eV, 285.88 eV, and 288.78 eV, corresponding to the C-C sp^2^, C-O, and O-C=O chemical bonds, respectively [53,58]. Figure 2f represents the deconvoluted XPS spectra of O 1s in the aerogel. The binding energies at 531.28 eV, 532.59 eV, 533.48 eV, and 535.37 eV are related to M-O, C-O, C-O=C, and C=O, respectively. These unique peaks demonstrate the successful formation of the aerogel with reduced graphene oxide (rGO). The presence of rGO dramatically improves the electrical conductivity of the aerogel, leading to an enhanced electrochemical performance. The XPS examination yielded valuable information about the chemical composition and bonding states of the elements in the M-rG-300 aerogel. This analysis validated the existence of MoS_2_ and rGO and demonstrated their effective interaction in the aerogel.

**Figure 3:**
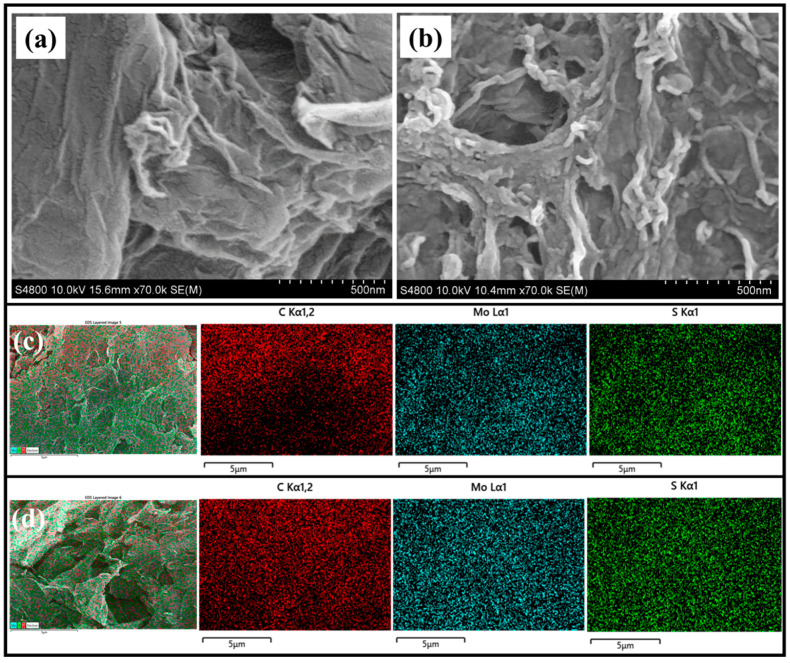
SEM micrograph images of (**a**) rGO and (**b**) M-rG300. EDAX mapping analysis results of (**c**) M-rG200 and (**d**) M-rG300.

The surface morphology of the as-prepared materials were investigated through field emission scanning electron microscopy (FE-SEM). Figure 3a and Figure 3b represent the FE-SEM images of rGO and the M-rG-300 aerogel, respectively. Figure 3a demonstrates the presence of a sheet-like architecture in the rGO. At the same time, the rGO was found to have crumbled, which is significantly different from the GO. As an indication that the rGO sheets have effectively reduced the amount of GO, a smooth surface and a low number of layers and folds are present in the sheets. It is possible to re-establish the sp^2^ carbon network via the removal of oxygen-containing functional groups, which ultimately results in an improvement in electrical conductivity [59]. The FE-SEM image of the as-developed M-rG-300 aerogel is represented in Figure 3b. The figures display that the aerogels showed an interconnected network-like structure with a 3D porous structure. This well-defined connectivity with porosity can effectively allow the easier penetration of electrolytes to all the catalytic sites. In addition to this, the nanorod-like MoS_2_ has been indicated to be successfully attached on the aerogel. This implies that during the hydrothermal process, the MoS_2_ formed on rGO, leading to the effective formation of the aerogel. The production of an outstanding aerogel is as a result of the efficient quantity of rGO combined with the MoS_2_, where multiple connections probably occur between the surface functional properties of each material. The excellent connectivity between the MoS_2_ and rGO can have an extraordinary impact on the electrochemical HER performances through a synergistic effect. EDAX was performed to identify the homogeneity of the aerogel. Figure 3c,d represent the EDAX mapping analysis of the M-rG200 and M-rG300 aerogels, respectively. Carbon, molybdenum, and sulfur elements have been observed in both aerogels. The results demonstrated a higher degree of homogeneity in the whole aerogel of the electrocatalyst. EDAX mapping results indicate that the aerogels have evenly dispersed MoS_2_ throughout the M-rG aerogel, which may have resulted in an increased catalytic activity.

**Figure 4:**
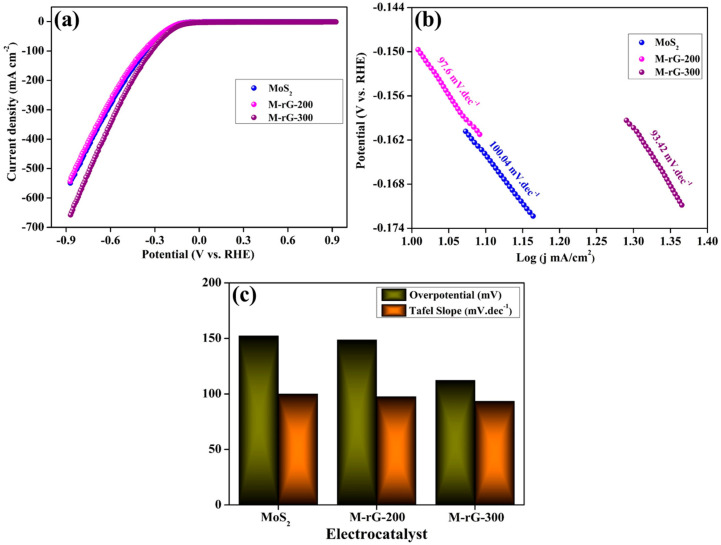
Hydrogen evolution reaction performance measurement results of MoS_2_, M-rG200, and M-rG-300. (**a**) LSV cures at 5 mV/s, (**b**) Tafel slope, and (**c**) comparative overpotential and Tafel results.

To estimate the hydrogen evolution reactions for the as-developed electrocatalyst materials, the electrochemical performance was measured in a standard three-electrode setup using the linear sweep voltammetry (LSV) technique in an alkaline electrolyte condition. The LSV performance was measured in N_2_-saturated 1 M KOH electrolyte at 5 mV s^−1^. In order to examine the immediate influence of the aerogels, the newly developed materials were fabricated using a substrate without binder and conductive carbon materials. Before being compressed, the aerogels were spread out equally over the Ni foam. A Pt plate and Hg/HgO functioned as the counter and reference electrodes, respectively, while the Ni foam that contains the electrocatalyst functioned as the working electrode. The LSV polarization curves of electrocatalysts based on MoS_2_ and the aerogel are shown in Figure 4a. Figure 4a,c demonstrate that the overpotential measured for nano-rod like structure of MoS_2_ at a current density of 10 mA/cm^2^ is 153 mV. The HER performance (Volmer–Heyrovsky and Volmer–Tafel mechanism [60,61]) of MoS_2_ mainly depends on the structure, dimension, and modification of MoS_2_. It is interesting to note that the performance of the electrocatalyst is effectively boosted by the aerogels. As embodied in Figure 4a, the HER performance of aerogels is effectively increased compared to the MoS_2_ alone electrocatalyst. The achieved lower overpotential of the M-rG-200 and M-rG-300 aerogel electrocatalysts is 148 and 112 mV, respectively, when the current density is 10 mA/cm^2^. The enhanced performance of the aerogels may be primarily attributed to the synergistic impact resulting from the effective connectivity between rGO and MoS_2_ during the production of the aerogel. Among the two aerogels, M-rG-300 exhibited a superior performance in the HER response compared to M-rG-200. The lower overpotential of 112 mV at 10 mA/cm^2^ measured for M-rG-300 is much lower than that obtained for M-rG-200 under similar operating conditions. The M-rG-300 electrocatalyst has produced an outstanding aerogel formation in comparison to the M-rG-200 electrocatalyst. This is the primary cause for promoting and achieving efficient HER performances. The excellent aerogel formation leads to a higher performance because of the interconnected network-like structure with a high degree of homogeneity. It can be concluded that M-rG-300 showed a superior HER performance in comparison to the other electrocatalysts used in this investigation. In order to better understand the kinetics of the HER, the intrinsic characteristics of the electrocatalysts were determined based on the Tafel slope value, which is indicative of the rate-determining steps of the reaction. The Tafel slope values are determined as illustrated in Figure 4b. The Tafel slope value of M-rG-300 (93.4 mV.dec^−1^) is much lower than that of the MoS_2_ and M-rG-200 electrocatalysts. The evidence obtained from the Tafel slope demonstrates that M-rG-300 has an improved reaction kinetic for the HER. The synergistic effect of including rGO and MoS_2_ in the aerogel results in highly effective electrocatalysts due to the enhanced electrical conductivity and an increased number of electrocatalytic active sites. To achieve HER performance, the rGO in the M-rG-300 electrocatalyst contributes to efficient electron transportation and MoS_2_ contributes to the greater rate of the water dissociation process. Furthermore, the creation of a synergic effect in M-rG-300 is the most probable mechanism to boost the electrocatalytic activity via favorable mass-transfer routes, which is also a key reason for attaining an excellent HER activity.

**Figure 5:**
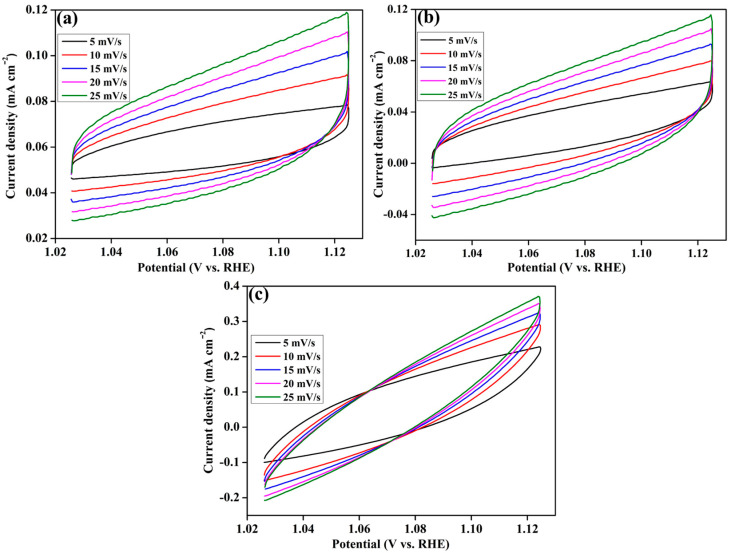
Cyclic voltammetry measurements at different scan rates: (**a**) MoS_2_, (**b**) M-rG200, and (**c**) M-rG300 electrocatalysts.

**Figure 6:**
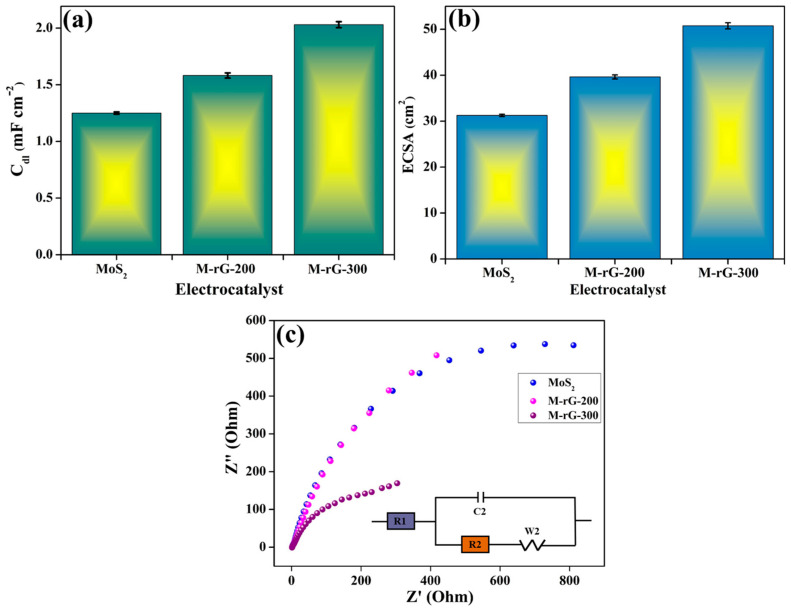
(**a**) C_dl_, (**b**) ECSA, and (**c**) EIS of MoS_2_, M-rG-200, and M-rG-300 electrocatalysts.

The performance of the electrocatalyst is linked to the electrochemical active surface area (ECSA) of the electrocatalyst. The ECSA measurement is used to validate the inherent electrochemical activity of the electrocatalysts. To evaluate the electrochemical double layer capacitance (C_dl_) of the electrocatalyst in the solid–liquid interface, cyclic voltammetry (CV) analyses were performed at different scan rates (5, 10, 25, 20, and 25 mV/s) using N_2_-saturated 1 M KOH electrolyte solution in the double-layer (non-faradaic) region. Figure 5a–c represents the CV curves of the MoS_2_, M-rG-200, and M-rG-300 electrocatalysts at various scan rates for identifying the number of active sites. As compared to the CV curves of MoS_2_ (Figure 5a) and M-rG-200 (Figure 5b), the area under the curves of CV is higher for M-rG-300. Based on the CV results, the C_dl_ (solid–liquid interface) of the electrocatalyst was determined via the function of scan rate. As a result of analyzing the relationship between scan rate and ΔJ, the C_dl_ was calculated for the MoS_2_, M-rG-200, and M-rG-300 electrocatalysts. Among the different electrocatalysts, M-rG-300 exhibits higher C_dl_ values than MoS_2_ and M-rG-200. As represented in Figure 6a, the obtained C_dl_ values are 1.25, 1.605, and 2.05 mF/cm^2^ for the MoS_2_, M-rG-200, and M-rG-300 electrocatalysts, respectively. The C_dl_ data show a direct correlation with the ECSA of the electrocatalysts. For determining the ECSA, the specific capacitance in an alkaline medium was considered [62]. The following equation was used to calculate the ECSA [63]:

ECSA = C_dl_/C_s_

The ECSA acquired a similar trend as the results of the C_dl_ were accomplished. Figure 6b represents the ECSA of the MoS_2_, M-rG-200, and M-rG-300 electrocatalysts. Compared to the MoS_2_ and M-rG-200 elecrocatalysts, the M-rG-300 electrocatalyst with the most excellent ECSA value is most impressive. Comparatively, the ECSA that was achieved for M-rG-300 is nearly two-fold greater than that of MoS_2_. Moreover, M-rG-300 also showed a 27.7% increase in ECSA compared to M-rG-200. The ECSA findings indicate that the optimal combination of rGO and MoS_2_ in an aerogel resulted in a large number of active sites with suitable porosity. This effectively impacts the HER process by providing a greater number of surface sites for reactions. The electrochemical impedance spectroscopy (EIS) method was used to assess the charge transfer ability in the electrocatalyst materials. The Nyquist plots of the MoS_2_, M-rG-200, and M-rG-300 electrocatalysts are represented in Figure 6c. The equivalent circuit is provided in the insert image in Figure 6c. It has been observed that the charge transfer resistance of the M-rG-300 electrocatalyst is much lowered when compared to the MoS_2_ and M-rG-200 electrocatalysts. Relatively, this phenomenon suggests that an efficient charge transfer occurs between the electrolyte and M-rG-300. The excellent aerogel formation, increased interconnected network-like structure, and efficient electrical conductivity by sufficient rGO in the M-rG-300 are the major reasons for obtaining the increased charge transfer compared to the M-rG-200 electrocatalyst. The HER performances are significantly impacted by the efficient electrical conductivity and diffusion channels in M-rG-300.

**Figure 7:**
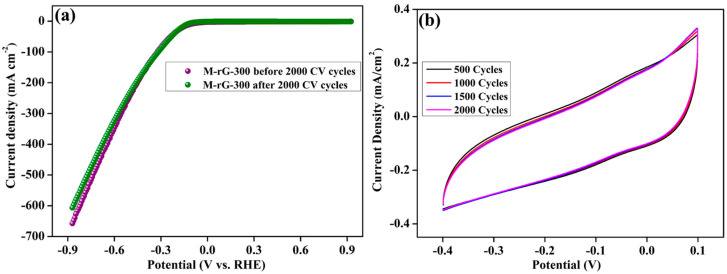
Stability measurements of the M-rG-300 electrocatalyst. (**a**) LSV analysis at a scan rate of 5 mV s^−1^ and (**b**) CV measurement at a scan rate of 50 mV s^−1^.

The stability of the M-rG-300 electrocatalyst (Figure 7a) was determined using LSV measurements before and after 2000 cycles of the CV test. Initially, the LSV measurement (Figure 7a) was carried out at a scan rate of 5 mV s^−1^, and then the CV analysis was measured for nearly 2000 cycles at a scan rate of 50 mV s^−1^ (Figure 7b). After completing the 2000 CV cycles, the LSV performance was measured at a scan rate of 5 mV s^−1^ and was compared with the initial LSV cycle, as represented in Figure 7a. Based on the LSV analysis, the M-rG-300 electrocatalyst exhibited a consistent and reliable performance both before and after conducting CV cycles. However, a modest decrease in performance was seen after the CV cycles, which may be due to the detachment of a small quantity of electrocatalyst material from the Ni foam. Excellent stability was found by further modifying the concept of the M-rG-300 electrocatalyst as a free-standing electrode. M-rG-300 electrocatalysts had a superior HER performance when compared to M-rG-200 electrocatalysts. Specifically, M-rG-300 electrocatalysts demonstrated a lower overpotential and a greater ECSA. The enhanced performance of the rG-300 aerogels is primarily attributed to the presence of a synergistic effect, which is facilitated by the effective connectivity between the rGO and MoS_2_ during aerogel production. The formation of a light density of a 3D porous structure of interconnected network-like M-rG-300 aerogels can provide a high specific surface area. This phenomenon can increase the large number of active sites and enhance/ease the diffusion pathways, which effectively improves the HER activity. The presence of rGO in the M-rG-300 electrocatalyst facilitates efficient electron transport, while MoS_2_ enhances the reaction rate for attaining efficient HER performances. The current study has the potential to be expanded to include different dimensionalities, phases, and crystallinities of MoS_2_ structures, surface modification of MoS_2_, and free-standing electrode concepts based on MoS_2_-containing rGO hybrid aerogels.

## 3. Conclusions

Here, we reported the successful fabrication of a MoS_2_-rGO hybrid aerogel as an effective electrocatalyst for the HER, consisting of a nanorod-like structure of MoS_2_ coupled with GO. The synthesis process was carried out according to the following two-steps: (i) the preparation of graphene oxide (GO) and (ii) the preparation of the M-rG hybrid aerogel. The well-defined and interconnected network-like structure M-rG hybrid aerogel was created using a saturated NaCl solution-assisted process via a hydrothermal technique. Furthermore, the comprehensive structural and microstructural investigations have confirmed the homogeneity of the aerogel and its effective creation. Compared to M-rG-200, M-rG-300 performed very well in relation to its ability to produce an aerogel. At a current density of 10 mA/cm^2^, the M-rG-300 aerogel had a significantly minimized overpotential of 112 mV compared to M-rG-200 (148 mV). A significant difference can be seen between the Tafel slope value of the M-rG-300 electrocatalyst (93.4 mV.dec^−1^) and those of the MoS_2_ and M-rG-200 electrocatalysts. According to the ECSA results, the aerogels with the ideal ratio of rGO to MoS_2_ produced many active sites. The exposure of greater active sites for the HER, electrical conductivity, and achieving a larger electrochemical active surface area were all impacted by the excellent connectivity between the MoS_2_ and rGO in the hybrid aerogel. Aerogel electrocatalysts made with rGO and MoS_2_ have a synergistic effect that boosts electrical conductivity and increases the number of electrocatalytic active sites, making a very effective electrocatalyst for the HER. It was also demonstrated that the M-rG-300 aerogel electrocatalyst exhibited greater stability.

## 4. Materials and Methods

Graphite (99.9%, Alfa Aesar, Ward Hill, MA, USA), ammonium molybdate tetrahydrate (H_24_Mo_7_N_6_O_24_, extra pure, Fisher chemical, Loughborough, UK), and sodium chloride (NaCl, ≥99.0%, Sigma Aldrich, St. Louis, MI, USA) were purchased and used in the present study. Sulfuric acid (H_2_SO_4_, ~95%), orthophosphoric acid (H_3_PO_4_, 98%), potassium permanganate (KMnO_4_, ~99.3%), hydrogen peroxide (H_2_O_2_, 30%), and thiourea (CH_4_N_2_S, 95%) were obtained from Duksan Chemicals and Metal, Hwaseong, Republic of Korea. Absolute ethyl alcohol (C_2_H_5_OH, 99.9%) and potassium hydroxide (KOH, >85%) were purchased from Daejung Chemicals & Metals, Siheung-si, Republic of Korea. De-ionized water (diH_2_O) was used in the present investigations. Nickel foam was acquired from NARA cell-Tech Corporation, Seoul, Republic of Korea.

The aerogel was synthesized following a two-step process: firstly, the conversion of graphite to graphene oxide (GO), and secondly, the transformation of GO to reduced graphene oxide (rGO) with MoS_2_ as hybrid aerogels (M-rG aerogel). The as-received bulk-sized graphite was initially ball-milled to reduce the size/layers. Improved synthesis methods were used to prepare the GO [49]. An anticipated amount of graphite was added to the 1:9 ratio of H_3_PO_4_:H_2_SO_4_ (180 mL:20 mL) mixture solution and was then sonicated and stirred for 20 min. Afterward, KMnO_4_ (9 g) was slowly added to the graphite containing H_3_PO_4_:H_2_SO_4_ solution under continued stirring. After entirely adding KMnO_4_, the solution was stirred for 12 h at 50 °C. After cooling the solution to room temperature, it was added to the 200 mL of ice prepared with 1.5 mL of 30% H_2_O_2_. Next, the as-prepared GO was washed with diH_2_O, 30% HCl, and ethanol. Finally, the as-developed product was washed several times with diH_2_O until reaching a neutral pH. The GO was dried at room temperature using a desiccator. For the comparative structural measurements, the rGO was developed by the addition of hydrazine hydrate and ammonia in GO solution via the hydrothermal method at 160 °C for 24 h. A saturated NaCl solution was utilized for creating the nanorod-like structure of MoS_2_ [50] in the aerogel. For devolving the M-rG aerogel, an anticipated amount (200 or 300 mg) of GO was dispersed in the 40 mL of saturated NaCl solution using a probe sonication technique. Afterward, 1 mmol of H_24_Mo_7_N_6_O_24_ was added to the GO-containing solution. Afterward, CH_4_N_2_S was added and stirred for 30 min. The as-prepared solution was further transferred into the 50 mL Teflon-lined stainless steel autoclave to undergo a hydrothermal process. Initially, the reaction was maintained at 80 °C for 30 min and then at 180 °C for 24 h. After the reaction was completed, the as-developed aerogel was carefully removed from the reactors and was cautiously treated with diH_2_O for solvent exchange. The as-prepared aerogel was freeze-dried in FD-1000, EYELA, Tokyo, Japan, for over 48 h. The 200 and 300 mg of GO were used for developing the M-rG aerogel, denoted as M-rG-200 and M-rG-300, respectively.

The X-ray diffraction (XRD, Xpert Pro) technique was utilized to identify the crystalline properties and structural information of the as-prepared materials. The chemical structure and molecular states of the electrocatalysts were verified through Raman spectroscopy (XploRA plus, HORIBA Jobin Yuon S.A.S, Longjumeau, France). To identify the chemical composition and oxidation states, the X-ray photoelectron spectroscopy (XPS, Thermo Scientific ESCALAB 220iXL, Waltham, MA, USA) technique was utilized. Field emission scanning electron microscopy (FE-SEM with EDAX, Hitachi S-4800, Tokyo, Japan) was used to determine the microstructure of the electrocatalysts. Energy-dispersive X-ray analysis (EDAX) was used to measure the elemental composition in the electrocatalysts.

In order to test the electrochemical performance, a typical three-electrode setup was used in the Corrtest electrochemical workstation (CS350 in COM3). For developing the working electrode, nickel (Ni) foam was considered. At first, the Ni foam was cut into 1 × 1 cm; pre-treated in 3 M HCl, DI water, and EtOH for 20 min in each; and dried. Afterward, a desired amount of as-developed electrocatalyst materials were spread on the surface of the nickel foam. Hydraulic compressed process were used to prepare the working electrodes, where the packing density of the aerogel increased in the substrate. Furthermore, Hg/HgO and a Pt plate were used as the reference and counter electrodes, respectively. For measuring the electrochemical performances, N_2_-saturated 1 M KOH solution was used as an electrolyte. Linear sweep voltammetry (LSV) was performed at a scan rate of 5 mV s^−1^ to identify the electrocatalysts HER activity. The following Nernst equation was used to determine the potential vs. reversible hydrogen electrode (V vs. RHE)

E_RHE_ = E_Hg/HgO_ + 0.0591 × (pH) + E^°^_Hg/HgO_

To determine the electrochemical active surface area (ECSA) of the electrocatalyst, cyclic voltammetry (CV) tests were carried out at several scan rates (5, 10, 25, 20, and 25 mV/s) using a N_2_-saturated 1 M KOH electrolyte solution in the double-layer (non-faradaic) region. The electrochemical impedance spectroscopy (EIS) technique was verified in the frequency range of 100 kHz to 0.1 Hz using a 10 mV amplitude to evaluate the charge transfer capability of the electrocatalyst materials. LSV measurements determined the stability of the optimized electrocatalyst before and after 2000 cycles of CV (50 mV s^−1^).

## Data Availability

The original contributions presented in this study are included in the article; further inquiries can be directed to the corresponding author.
